# Supplementary material for: Indirect associations of pain resilience and kinesiophobia with the relationship between physical activity and chronic pain
Source: PLoS One. 2025 Oct 22;20(10):e0334144. doi: 10.1371/journal.pone.0334144 (PMC12543166; doi:10.1371/journal.pone.0334144)
Supplement: Attachment — Submitted filename: Response to Reviewers.docx [file pone.0334144.s002.docx]

**Journal Requirements:**

Please review your reference list to ensure that it is complete and correct. If you have cited papers that have been retracted, please include the rationale for doing so in the manuscript text, or remove these references and replace them with relevant current references. Any changes to the reference list should be mentioned in the rebuttal letter that accompanies your revised manuscript. If you need to cite a retracted article, indicate the article’s retracted status in the References list and also include a citation and full reference for the retraction notice.

**We have checked the reference list following the journal’s advice and have not identified any issues.**

Editor Comments

1. Clarify in the text that the findings represent associations rather than cause-and-effect relationships, and strongly recommend future longitudinal or experimental studies.

**We have made the suggested changes and clarified in the text that the findings are correlational in nature and highlighted the need for further longitudinal and experimental investigation. See for example, pg. 15, ln. 5-6.**

2. The sample is extremely heterogeneous, including individuals with different chronic pain conditions, without distinguishing between types of pain. The psychometric tools used were validated for specific populations but were applied indiscriminately to a general chronic pain population. This point should be emphasized in the discussion as a critical limitation, warning that the results may not be uniformly applicable to all chronic pain conditions.

**This has been further emphasised in the limitations sections as suggested.**

3. A more balanced discussion is advised, acknowledging the fragility of some findings and avoiding excessive generalizations regarding the clinical applicability of the results.

**We have adjusted the discussion section in line the suggestions.**
