# Supplementary material for: Indirect associations of pain resilience and kinesiophobia with the relationship between physical activity and chronic pain
Source: PLoS One. 2025 Oct 22;20(10):e0334144. doi: 10.1371/journal.pone.0334144 (PMC12543166; doi:10.1371/journal.pone.0334144)
Supplement: Attachment — Submitted filename: Response_to_Reviewers_auresp_3.docx [file pone.0334144.s004.docx]

**Journal Requirements:**

Please review your reference list to ensure that it is complete and correct. If you have cited papers that have been retracted, please include the rationale for doing so in the manuscript text, or remove these references and replace them with relevant current references. Any changes to the reference list should be mentioned in the rebuttal letter that accompanies your revised manuscript. If you need to cite a retracted article, indicate the article’s retracted status in the References list and also include a citation and full reference for the retraction notice.

**We have checked the reference list following the journal’s advice and have not identified any issues.**

Reviewer 4

Thank you for the opportunity to review this interesting and timely manuscript. The authors address an important topic by examining the psychological mechanisms underlying the relationship between chronic pain and physical activity, with a focus on pain resilience and kinesiophobia. This is a relevant and clinically meaningful contribution, as understanding protective and maladaptive psychological factors can inform both prevention and intervention strategies.

With minor revisions to clarify methodology, streamline the narrative, and strengthen the discussion of limitations and clinical implications, this paper has the potential to make an important impact in the field.

**Thank you for your kind words and encouragement. We have incorporated your suggestions and feel this has greatly improved the manuscript.**

Abstract

Some sentences are overly long and complex (e.g., “Significant proportions of the association between musculoskeletal pain on physical activity were found to occur…”). Shorter, more direct sentences would improve clarity.

Consider rephrasing “on the relationship between pain and physical activity” to “in the relationship between pain and physical activity”.

The abstract mentions “three models” but does not specify what type (e.g., mediation analysis, path models). A brief clarification would enhance transparency.

The conclusion is strong but slightly overstated. For balance, add a qualifier about study design (e.g., “Given the cross-sectional design, longitudinal studies are needed to confirm causal pathways”).

Consider emphasizing the potential clinical application of fostering resilience (e.g., through behavioral or psychological interventions).

**Thank you for your suggestions. We have adapted the abstract accordingly.**

Introduction

The introduction provides a strong conceptual framework and justification for the study, but it would benefit from improved organization, reduced redundancy, and a sharper articulation of the study aim. With minor revisions, it can effectively set up the research question and highlight the study’s contribution.

Minor comments.

Some sentences repeat concepts (e.g., multiple mentions of kinesiophobia leading to activity avoidance). Streamlining these sections would improve readability.

Paragraphs are somewhat long and could be broken into smaller sections to improve clarity, especially the sections on kinesiophobia and pain resilience. For example, break the introduction into three focused paragraphs: (1) physical activity and chronic pain, (2) negative psychological factors (kinesiophobia), and (3) positive psychological factors (pain resilience) leading to the study aim.

The introduction sometimes shifts between broad chronic pain populations and specific conditions (e.g., osteoarthritis, fibromyalgia, low back pain). Consider clearly specifying whether the study targets a particular population or general chronic pain sufferers.

The transition from negative psychological factors (kinesiophobia) to positive factors (pain resilience) is slightly abrupt. A bridging sentence explaining why examining positive factors complements existing research would improve flow. Smoothly transition from negative to positive psychological factors, emphasizing how examining both provides a fuller understanding of the pain–activity relationship.

A few sentences are slightly wordy or complex. For example, “It is, however, not clear if this extends to affecting the association between pain and physical activity” could be simplified to “It remains unclear whether this also influences the pain–physical activity relationship.”

Consider highlighting the novelty or practical significance of investigating pain resilience as a modifiable factor.

**Thank you for your suggestions to improve our introduction. We have actioned all your suggestions with one exception, please see below.**

The final sentence introduces the study aim but could be more concise and explicit. For example, clearly stating: “This study aims to investigate whether pain resilience mediates the relationship between chronic pain and physical activity” may strengthen clarity.

**We specifically decided not to label this as mediation, as the term mediation implies a causal relationship that we can not infer given the limitations of the cross-sectional study design.**

Methods

The Methods section is thorough and provides sufficient detail for replication. However, improvements in organization, conciseness, and clarity would enhance readability and focus. With minor revisions, this section would clearly communicate the study design, participants, measures, and statistical approach to the reader.

Minor inconsistencies in percentages (e.g., 86 females (50.3%) vs. 83 males (48.3%) with 2 non-binary/third gender (1.2%) and 1 not reported (0.6%) adds to >100%) should be double-checked for accuracy. Ensure all percentages and participant characteristics add correctly to 100%.

**Thank you for pointing out this oversight. 86 out of 172 is indeed 50.0%. We have amended this in the manuscript. Please note, the numbers still do not add up to exactly 100%, but this is due to rounding to one decimal.**

While all measures are described, the rationale for including some (e.g., FRAIL scale) in this context could be made clearer. How does frailty relate to chronic pain, resilience, or physical activity in this study? Provide a brief justification for including the FRAIL scale and its relevance to the study outcomes.

**Frailty reflects vulnerability to adverse health outcomes, which are highly relevant in the context of chronic pain** ^1^**. Frailty has been linked to higher pain prevalence, lower resilience, and reduced capacity for physical activity, making it a meaningful construct for understanding variability in pain experiences and rehabilitation outcomes in this population. We have added a brief justification to the manuscript and a reference to** ^1^**.**

The section could briefly mention whether assumptions for the statistical models (e.g., normality, linearity) were checked.

**Thank you for suggesting this. We have reported assumptions testing in the statistical methods section, e.g., assessing the normality of the residuals.**

Discussion

The text repeatedly notes that mechanisms are “unclear” or “cannot be ruled out.” Stronger, more specific hypotheses or a conceptual model would add value.

**Thank you for your suggestion. The cautious language was requested by previous reviewers and editors to highlight the fact that causality cannot be established in the current study.**

The gender-related findings are mentioned but cultural explanations are very brief. More evidence or references on cultural and social aspects would strengthen the argument.

The overlap between frailty and pain resilience is only briefly mentioned. A deeper discussion or concrete suggestion for future research would improve this section.

Several grammatical errors and awkward phrases (e.g., “kinesiophobia was not contribute significant unique variance”) reduce readability. Careful English editing is needed.

The section ends with limitations but lacks a strong concluding paragraph summarizing the central findings, clinical relevance, and need for longitudinal studies.

**We have actioned all your suggestions. We have added an additional reference** ^2^**.**

**References:**

1.	Lin, T., Zhao, Y., Xia, X., Ge, N. & Yue, J. Association between frailty and chronic pain among older adults: a systematic review and meta-analysis. *Eur Geriatr Med* **11**, 945–959 (2020).

2.	Mathew Joseph, N., Ramaswamy, P. & Wang, J. Cultural factors associated with physical activity among U.S. adults: An integrative review. *Applied Nursing Research* **42**, 98–110 (2018).
